# Orai3 mediates Orai channel remodelling to activate fibroblast in pulmonary fibrosis

**DOI:** 10.1111/jcmm.17516

**Published:** 2022-09-20

**Authors:** Changhui Yu, Zicong Zhou, Wufeng Huang, Xiumei Li, Fei Zou, Xiaojing Meng, Shaoxi Cai

**Affiliations:** ^1^ Chronic Airways Diseases Laboratory, Department of Respiratory and Critical Care Medicine, Nanfang Hospital Southern Medical University Guangzhou China; ^2^ Guangdong Provincial Key Laboratory of Gastroenterology, Department of Gastroenterology, Nanfang Hospital Southern Medical University Guangzhou China; ^3^ Guangdong Provincial Key Laboratory of Tropical Disease Research, Department of Occupational Health and Occupational Medicine, School of Public Health and Tropical Medicine Southern Medical University Guangzhou China

**Keywords:** fibroblast, Orai3, pulmonary fibrosis, SEPTIN4, SOCE

## Abstract

Orai family are a calcium channel of cell membrane extracellular Ca^2+^ influx which participates in tissue fibrosis. But the roles of Orai3 have less attention on the mechanism of regulating lung fibrosis. In this study, we found that Orai3 expression was increased significantly in BLM‐induced lung fibrosis. The knockdown of Orai3 decreased TGF‐β1‐induced fibroblast proliferation, ECM production, activation of NFAT1 and Calpain/ERK signal pathway and glycolysis levels. Orai3 interacting with Orai1 was increased in BLM‐induced lung fibrosis and TGF‐β1‐induced fibroblast, while the Stim1 interacting with Orai1 and SOCE activity was suppressed, leading in a high and stable extracellular Ca^2+^ influx. Furthermore, the over‐expression of Orai3 did not enhance Orai3 interacting with Orai1 under TGF‐β1 free fibroblast. And then, the deeper mechanism of TGF‐β1‐induced increased SEPTIN4 promoted Orai3 interacting with Orai1. Our results indicated that Orai3 could be one of the therapy targets for PF in which remodels Orai channel, suppresses SOCE activity and activated fibroblast to alleviate fibrosis progress.

## 1 INTRODUCTION

Pulmonary fibrosis (PF) is a progressive, chronic lung disease with a poor prognosis and limited treatment options and characterized by alveolar epithelial cells damage and the subsequent proliferation of activated lung fibroblasts and myofibroblasts.^1^, ^2^ Fibroblast activation plays an important role for IPF, the activated fibroblast producing excessive accumulated extracellular matrix (ECM) proteins resulting in damaged gas exchange and impaired pulmonary function,^3^, ^4^ but the underlying mechanism of the activation of fibroblast is not completely clear yet.

Ca^2+^ is one of the second messengers in intracellular signalling pathways, there are plenty of evidences recently showed that Ca^2+^ influx promotes activation of fibroblast. Store‐operated Ca^2+^ entry (SOCE) is a critical mechanism of Ca^2+^ influx in non‐excitable cells. Stromal interaction molecule1 (STIM1) and Orai1 are responsible for SOCE, which was activated by Ang II in cardiac fibroblasts led to fibrotic cascade in cardiac fibrosis.^5^, ^6^ Moreover, Orai1 could form Ca^2+^ influx channel by store independent Ca^2+^ entry (SICE)^7^ and Orai3 plays the role of a partner for Orai3‐Orai1 heteromultimeric channels.^8^, ^9^ Interestingly, the reports have demonstrated that the formation of Orai3‐Orai1 heteromultimeric channels was determined for cancer cells proliferation and anti‐apoptosis.^8^ However, the role of Orai3 on lung fibrosis is not clear.

In this study, we demonstrated that Orai3 mediated TGF‐β1‐induced fibroblast proliferation, ECM production, activation of NFAT1 and Calpain/ERK signal pathway and glycolysis levels. The formation of Orai3‐Orai1 heteromultimeric channels increased in fibrotic models and remodelled Orai channel, leading in SOCE activity suppress and a high and stable extracellular Ca^2+^ influx. And then, the deeper mechanism of increased SEPTIN4‐induced Orai3 interacting with Orai1 to remodel Orai channel. Our results revealed a new mechanism by which Orai3‐Orai1 heteromultimeric channels activates fibroblasts to develop pulmonary fibrosis.

## 2 MATERIALS AND METHODS

### 2.1 Animals

All the animal experiments were performed by the Committee on the Ethics of Animal Experiments of Southern Medical University in Guangzhou, China, and applied under guidelines for the Care and Use of Laboratory Animals. Specific‐pathogen‐free Wistar rat (male, 4–6 weeks old) were purchased from Guangdong Medical Experimental Animal Center. The mice were housed in the Specific pathogen Free (SPF) with a 12 h light/dark cycle at 24°C room temperature and atmosphere of 40%–70% humidity. Food and water were sterilized. Twenty rats were randomly divided into two groups (*n* = 10 per group): the control, Bleomycin (BLM) groups. After anaesthesia, the rats in the BLM groups were conducted intratracheal injection of 200 μl BLM sulphate (5 mg/kg) to induce pulmonary fibrosis, while the control groups were intratracheally injected with equal volume of sterile saline. Twenty one days later, all the animals were sacrificed and the lungs were removed for further analysis.

### 2.2 Primary lung fibroblasts culture and treatments

Rats were used to isolate primary lung fibroblasts for experiments in vitro. Rats' lung was removed and minced with scissors in sterile PBS. And then, the minced lung tissues were transplanted to the bottom of T25 flasks with DMEM, containing 15% FBS and 2% penicillin–streptomycin, placed upside down for incubating 3 h at 37°C with an atmosphere of 5% CO^2^. After the incubation, turn the flasks up for continuing primary lung fibroblasts culture up to 80% confluence for the following experiments.

### 2.3 Pulmonary histologic examination

Left lungs from Section 2.1 were gently infused with 4% neutral formalin to fully inflate all lobes and immersed in formalin for at least 48 h, then fixed, paraffin‐embedded, cut in 5 μm sections and stained with haematoxylin and eosin and Masson for histopathologic detection. The paraffin sections of rats' lung tissues were collected from pathology department for routine immunohistochemistry (IHC) staining for anti‐Orai3, anti‐CRACR2A and anti‐SEPTIN4. The expression levels of Orai3, CRACR2A and SEPTIN4 in the tissue were scored according to the percentage of positive cells in each whole area of breast cancer tissue and their staining intensity as our previously described.^10^

### 2.4 Western blotting analysis

Cells or rat lung tissue were lysed in the RIPA lysis buffer (KeyGEN Biotech) containing PMSF (KeyGEN Biotech) at 4°C, 15 min. After ultrasonicated 10 min, the samples were centrifuged at 13,684.32 *g* for 10 min, and the supernatants were collected and boiled with standard SDS sample buffer. The samples were resolved by SDS‐PAGE, and Western blotting analysis was performed for detection of the following antigens: p‐ERK1/2, ERK1/2, β‐actin, Histone (Cell Signalling Technology), Orai1, Orai2, Orai3, Collagen I, α‐SMA, STIM1 (Sigma‐Aldrich), NFAT1, Calpain, CRACR2A, SEPTIN4, STIMATE, GOLLI, SARAF and ORMDL3. After incubation with a secondary antibody, signal intensities were analysed by using the Odyssey infrared Image System (Licor).

### 2.5 Analysis of glucose uptake, lactate dehydrogenase activation and lactate production

Glucose uptake levels were determined by measuring the uptake of 2‐NBDG according to the manufacturer's instructions (Keygen Biotech). Lactate dehydrogenase (LDH) activities and lactate production levels were determined with an LDH assay kit according to the manufacturer's instructions (Jiancheng Bioengineering Institute).

### 2.6 Intracellular level of C^a2+^ measurement

Intracellular level of Ca^2+^ was measured according to the recent reports.^11^ Briefly, the cells were loaded with 10 μmol/L Fluo4/AM for 30 min in dark and rinsed with D‐Hank for 30 min. SOCE analysis was performed using 2 μmol/L thapsigargin influx was initiated to empty Ca^2+^ in the endoplasmic reticulum. Ten minutes later, 2 mM CaCl2 was added to assess external Ca^2+^ influx. No‐SOCE analysis, 2 mM CaCl2 was added at first. The fluorescence signal was recorded and analysed by confocal laser scanning system (Leica). The changes in the peak value of cytosolic Ca^2+^ were displayed as a ratio of fluorescence relative to the baseline intensity before the application of TG or extracellular Ca^2+^ (F1/F0).

### 2.7 Protein interference or overexpression

Small interfering RNAs (siRNAs) targeting to the mRNAs of Orai1, Orai3 and SEPTIN or siRNA‐negative control (siNC), and plasmid for CRACR2A overexpression or negative control (NC) were purchased from Genepharma Co. and applied according to the manufacturer's instructions from lipo‐3000 (Life). Forty‐eight hours later, Western blot was used to detect the protein interference.

### 2.8 Co‐immunoprecipitation (Co‐IP) assays

Co‐immunoprecipitation assays were performed according to the instructions of Protein A/G Magnetic Beads for IP (Biotool). In brief, cell or lung tissue of rats lysates were prepared by using Lysis Buffer for Western and IP (Keygen) for Western and IP assays, then incubated with the Protein A/G Magnetic Beads‐Ab complex that were prepared in advance (5 μg Stim1 antibody plus 50 μl Protein A/G Magnetic Beads) at 4°C over‐night. The IP matrix–antibody complex was then washed with elution buffer (0.1–0.2 M Glycine, 0.1%–0.5% detergent, pH 2.5–3.1) and protein complexes were eluted and subjected to Western blotting assays.

### 2.9 Immunofluorescence staining (IF)

Cells were cultured on confocal dish fixed with 4% formaldehyde, permeabilized with 0.1% Triton X‐100 and blocked with 5% BSA for 10 min at 37°C. Cells were stained with primary antibody for a certain dilution, according to manufacturer's instructions, in 5% BSA and incubated overnight at 4°C, subsequently incubated with Alexa Fluor 488 (R37118) or Alexa Fluor 594 (R37119; 1:200 diluted in PBS; Invitrogen) at room temperature for 1 h protected from light and followed by incubation with DAPI (1:1000 diluted in PBS) for 5 min protected from light before laser scanning under a confocal microscope (FV1000, Olympus) at 100× objective magnification.

### 2.10 Cell proliferation

Cell proliferation was measured using the cell counting kit‐8 (CCK‐8, Beyotime) as previously described.^12^

### 2.11 Statistical analysis

Statistical analysis was carried out using the SPSS (version 19.0) software package. The variables were expressed as the mean ± standard deviation (SD). One‐way anova accompanied by Bonferonni's post hoc test for multiple comparisons were utilized to compare differences between groups. The values of *p* < 0.05 were considered to be statistically significant.

## 3 RESULTS

### 3.1 Orai3 is involved in fibroblast activation in lung fibrosis

**FIGURE 1:**
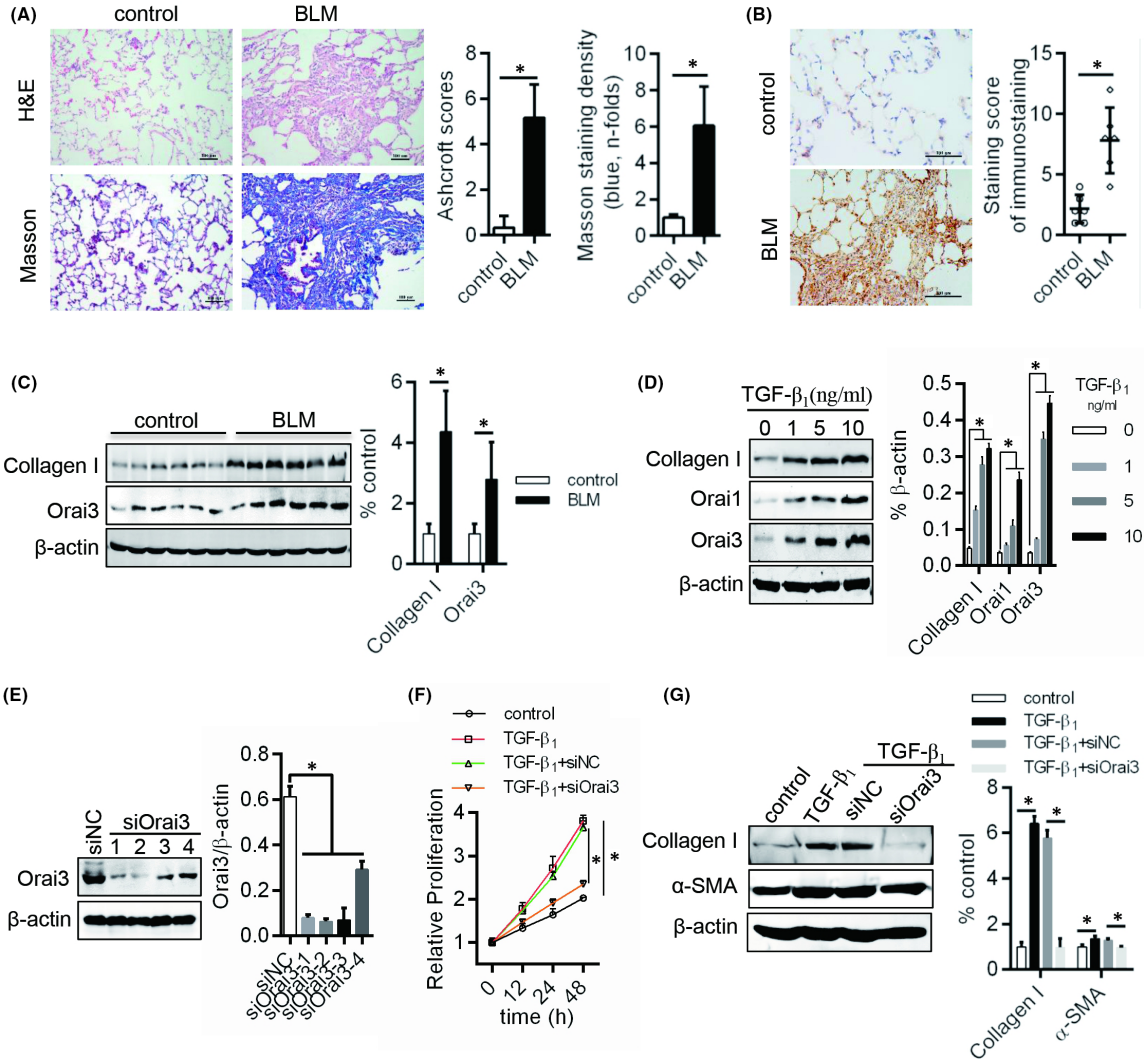
Effects Orai3 on fibroblast activation. (A) The lung fibrosis of rats was detected by haematoxylin and eosin (up) and Masson (down). The morphological changes in fibrotic lungs were quantified using the Ashcroft score. Measurement of the collagen area (blue) in the lungs of animals in designated treatment groups stained with Masson's trichrome. (B) Orai3 protein levels of lung in BLM‐managed rats was test by IHC. (C) The Collagen I and Orai3 protein levels of lung in BLM‐managed rats and TGF‐β1‐induced fibroblast was detected by Western blot. (D) siRNA for knocking down Orai3 was transfection into fibroblast, and then was verified by Western blot and siOrai3‐2 was used for following studies. (E) The potential of fibroblast proliferation was test by CCK‐8. (F) siRNAs were transfected into fibroblasts and the espression of Orai3 was detected by western blot. (G) Western blot was used to evaluate Collagen I and α‐SMA production of fibroblast. *p < 0.05

To determine whether Orai3 participate in lung fibrosis, fibrotic model in rat was induced by Bleomycin (BLM) mentioned in Methods. Haematoxylin and eosin staining and Masson staining were used to evaluate the pathological features in the fibrotic model lung tissues (Figure 1A). The fibrosis marker, Collagen I expression significantly increased in fibrotic rat lung tissue compared with control (Figure 1C). These data suggested that the fibrotic rat model was established successfully. Next, we further investigated that the expression of Orai3 was expectedly increase in BLM‐induced lung (Figure 1B,C). To determine whether the expression of Orai3 increased in vitro either, the primary lung fibroblasts were cultured and treated with TGF‐β1 (Figure 1D). The expression of Orai3 increased as well as the expression of Collagen I on concentration dependent of TGF‐β1 in primary lung fibroblast (Figure 1D). Next, we surprisingly found that the proliferation of primary fibroblast with TGF‐β1 treatment was decreased after interfering the expression of Orai3 by specific siRNA, and the expression of Collagen I and α‐SMA also decreased (Figure 1E,F). These data demonstrated that the Orai3 plays critical role in lung fibrosis in vivo or in vitro.

### 3.2 NFAT1 and Calpain/ERK signal pathway activation depended on Orai3 in TGF‐β1 treated‐ fibroblast

**FIGURE 2:**
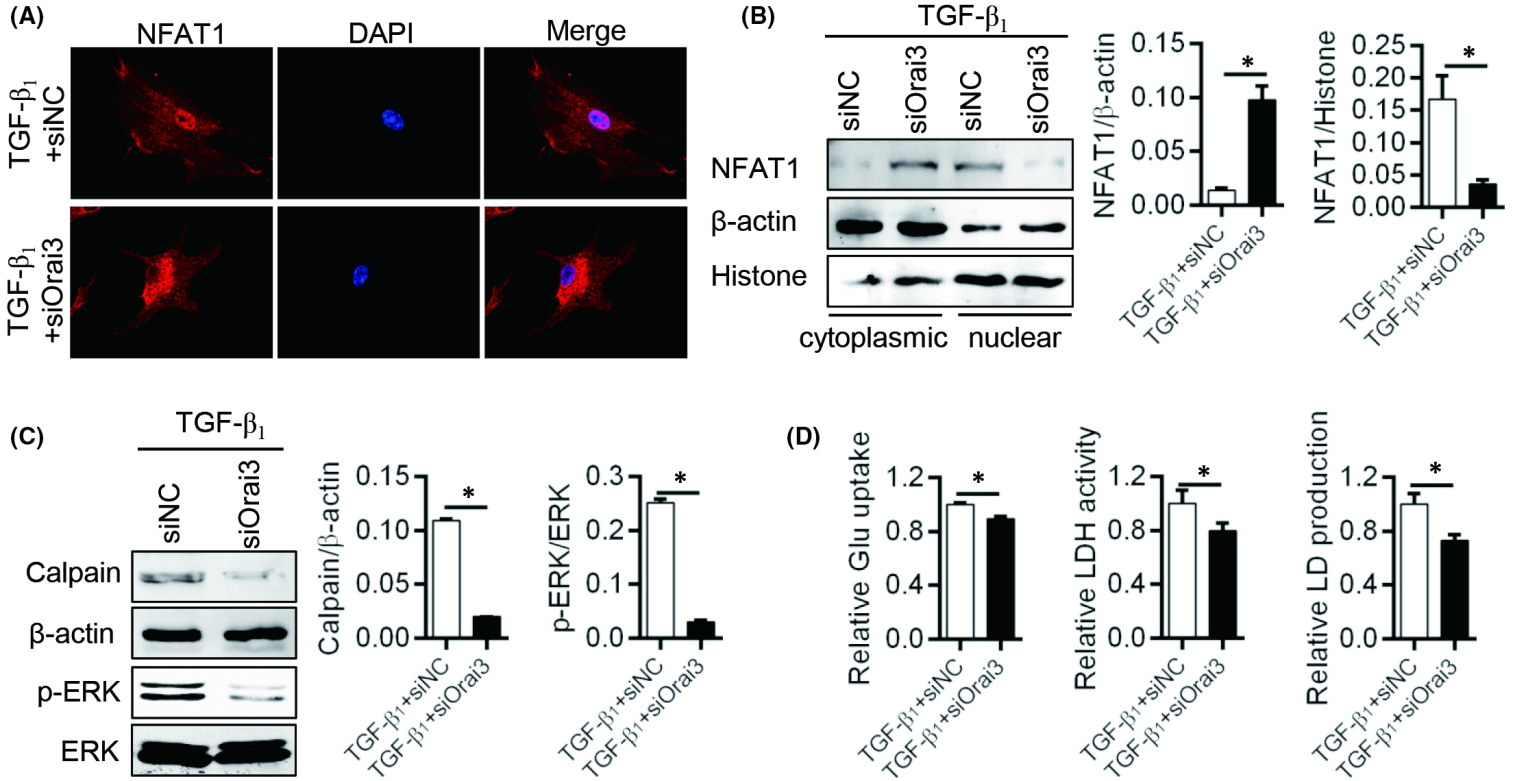
Orai3 mediated NFAT1 and Calpain/ERK signal pathway activation. (A) Immunofluorescence was used to assess the nuclear translocation of NFAT1 (red) in Orai3 knockdown or no fibroblast with TGF‐β1 treatment for 24 h, and the semi‐quantitative protein of NFAT1 in nuclear was run by Western blot, β‐Actin and histone were used as loading controls for the cytoplasmic and nuclear proteins, respectively (B). (C) The expression of Caplain and phosphorylated ERK was detected by Western blot. (D) Glucose uptake, LDH activity and lactate (LD) production in fibroblast expressing Orai3 knockdown with TGF‐β1 treatment for 24 h. *p < 0.05

Increased intracellular level of Ca^2+^ is required in which the calcineurin‐mediated dephosphorylation of the nuclear factor of an activated T cell (NFAT), and the NFAT1 is a Ca^2+^‐dependent downstream effector.^13^ We found that after knocked down Orai3 protein, the nuclear translocation of NFAT1 was significantly decreased in TGF‐β1 treatment fibroblasts (Figure 2A,B). And the expression of Calpain, a Ca^2+^ influx associated protein and phosphorylated ERK were also both decreased in Orai3 knockdown group (Figure 2C). Meantime, knocked down Orai3 expression also weakened the capacity of glucose uptake, affected activity of LDH and decreased production of LD to inhibit glycolysis which is an important pro‐fibrosis mechanism^14^ (Figure 2D). These data showed that Orai3 regulated Ca^2+^ influx to activating NFAT1 and Calpain/ERK signal pathway and increasing glycolysis in primary lung fibroblasts.

### 3.3 Orai channel remodelling suppressed SOCE

**FIGURE 3:**
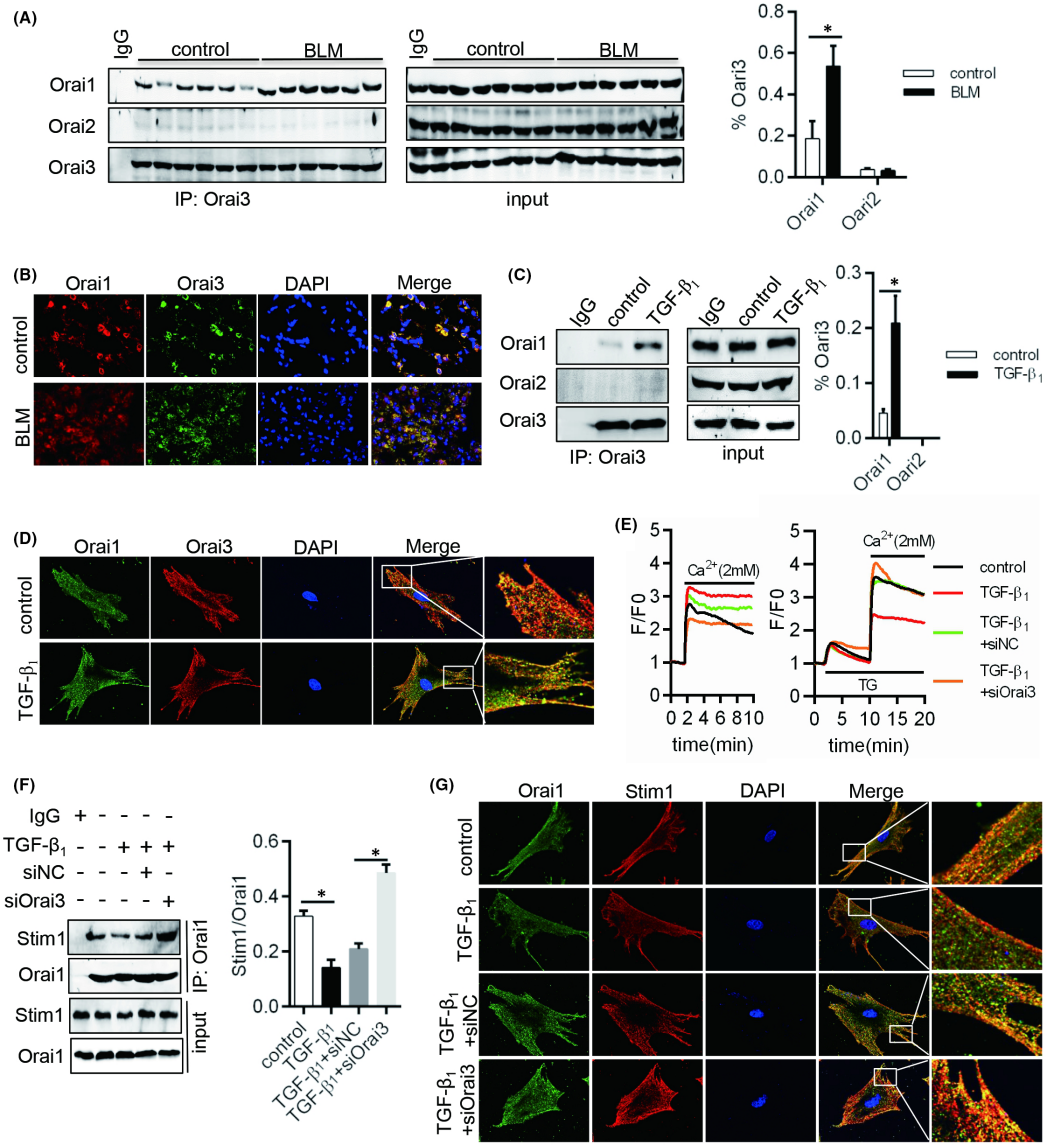
Orai remodelling suppressed SOCE. (A) Total lung tissue lysates from control and BLM group were Co‐IPed with an anti‐Orai3 antibody or control IgG. Orai1 and Orai2 levels are indicated. (B) Dual IF staining of Orai1 (red) and Orai3 (green) was performed to analyse formation of Orai1 and Orai3 complexin lung from BLM managed rats or no. Scale bars: 20 μm. The formation of Orai1 and Orai3 complex was detected by (C) Co‐IP and (D) dual IF staining of Orai1 (red) and Orai3 (green). Scale bars: 20 μm. (E) Comparison of no‐SOCE/SOCE activities, represented as Ca^2+^ peak amplitude in the F/F ratio over baseline values (F/F) after extracellular Ca^2+^ application. TG, thapsigargin. (F) Co‐IP and (G) dual IF staining of Orai1 (green) and Stim1 (red) was used to test colocalization of Orai1 and Stim1. Scale bars: 20 μm.*p < 0.05

Ca^2+^ release‐activated Ca^2+^ (CRAC) channels are formed by Orai1, Orai2 and Orai3 proteins and activated by stromal interaction molecule (Stim) 1 and Stim2. Among them, Stim1 binds and activates hexamers of Orai1 called store‐operated calcium (Ca^2+^) entry (SOCE).^15^ Recently, studies also reported that Orai remodelling was happened in cancer cell through Orai1 and Orai3 tetramer replacing Orai1 tetramer and did not need STIM1 activation. For our research, Orai3 interacted with Orai1 was significantly increased in lung from BLM‐induced fibrosis rat and fibroblast with TGF‐β1 treatment (Figure 3A–D). Beside, we found that the external Ca^2+^ influx (no‐SOCE) was enhanced and SOCE was decreased in TGF‐β1 treated‐fibroblast (Figure 3E), but the low expression Orai3 antagonized the effects of TGF‐β1 on no‐SOCE and SOCE (Figure 3E). Moreover, we observed that in spite of TGF‐β1 management fibroblast, the interaction of Stim1 and Orai1 was increased in Orai3 knockdown group (Figure 3F). These data demonstrated that Orai3 plays an important role for Ca^2+^ influx activation through interacting with Orai1 to remodel Orai channel and suppress SOCE activity in fibroblasts.

### 3.4 Orai3 promotes fibroblast activity depend on Orai1

**FIGURE 4:**
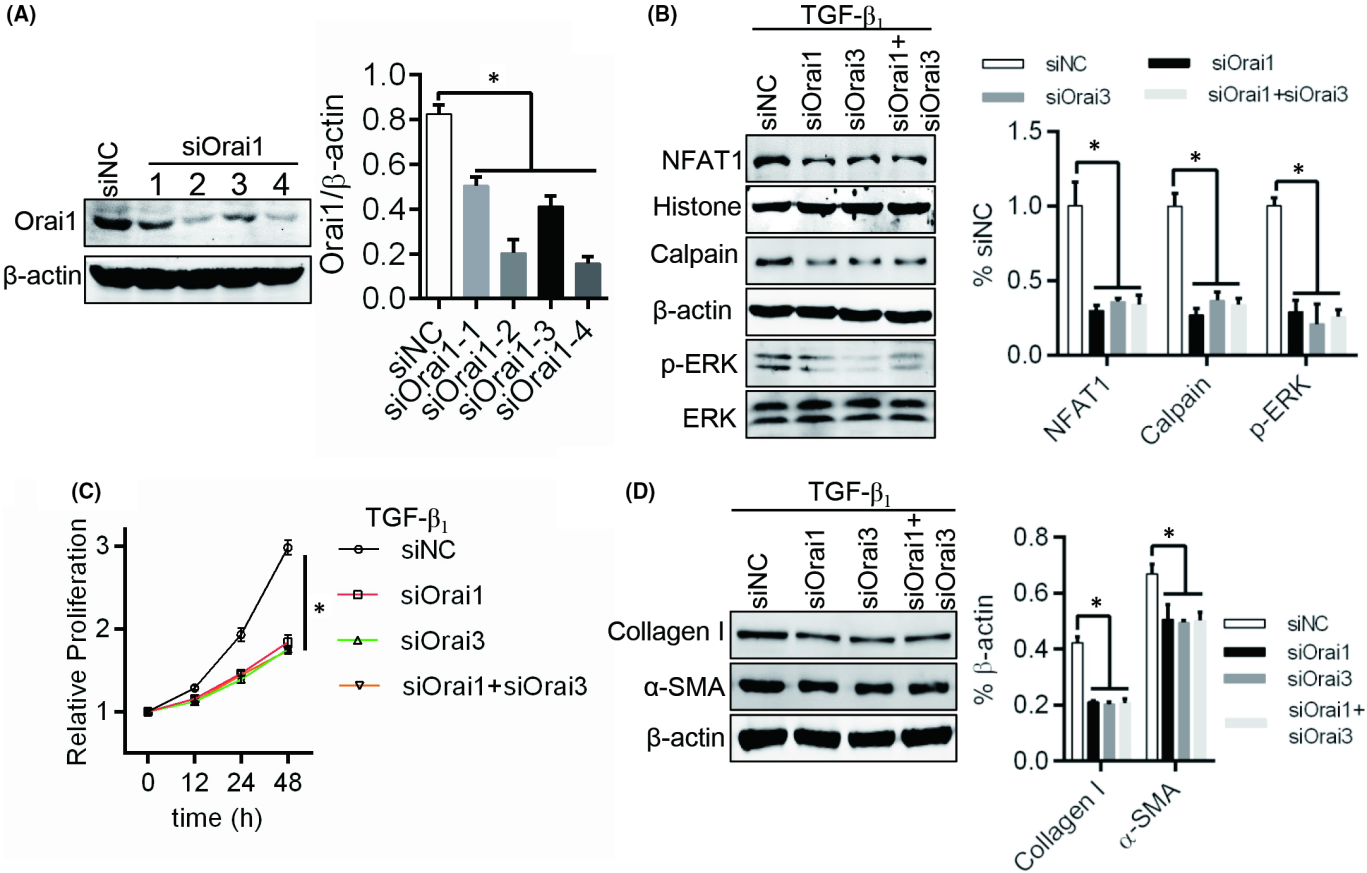
Orai3 promotes fibroblast activity depend on Orai1. (A) Orai1 expression was knocked down by siRNA and siOrai1‐4 was used for following studies. (B) The NFAT1 expression in nuclear, Caplain expression and phosphorylated ERK was detected by Western blot. β‐Actin and histone were used as loading controls for the cytoplasmic and nuclear proteins, respectively. (C) The potential of fibroblast proliferation was test by CCK‐8. (D) Western blot was used to evaluate ECM production of fibroblast with TGF‐β1 treatment or no. *p < 0.05

Thought Orai3 could interact with Orai1 to remodel Orai channel, whether the effects of Orai3 on fibroblast activity depend on Orai1. Firstly, the expression of Orai1 and Orai3 was knocked down, respectively, or together (Figure 4A). We found that the NFAT1 and Calpain/ERK signal pathway activation, cells proliferation and ECM production were suppressed in Orai1 low expression fibroblast under culture with TGF‐β1 (Figure 4B–D). And further knockdown of Orai3, above‐mentioned inhibition did not change (Figure 4B–D). Additionally, we observed the no‐SOCE and SOCE and found that the no‐SOCE was both decreased in Orai1 or/and Orai3 knockdown cells than that in control group (Figure S1). In SOCE, the Orai3 knockdown group was increased, but decreased in Orai1 knockdown cells (Figure S1). These data insinuate that Orai1 is required for Orai3 activating fibroblasts.

### 3.5 The increased expression of Orai3 is not the main pro‐remodelling factor of Orai channel

**FIGURE 5:**
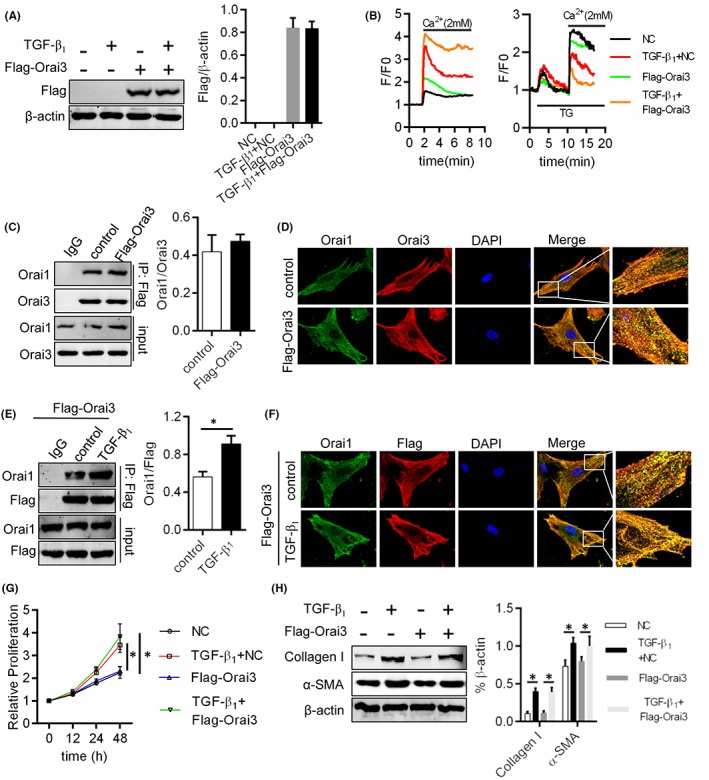
Effects of Orai3 expression levels on remodelling factor of Orai channel. (A) Plasmid expressing orai3 and carrying fusion gene flag was transfected into fibroblast and then TGF‐β1 managed for 24 h. Western blot was performed to test the flag and Orai3 expression. (B) Represented as Ca^2+^ peak amplitude in the F/F ratio over baseline values (F/F) after extracellular Ca^2+^ application for assessing no‐SOCE/SOCE activities. The formation of Orai1 and Orai3 complex was detected by (C) Co‐IP and (D) dual IF staining of Orai1 (green) and Orai3 (red). Scale bars: 20 μm. (E) Co‐IP and (F) dual IF staining of Orai1 (green) and flag (red) was used to assessed the interaction of Orai1 and flag‐Orai3. (G) The potential of fibroblast proliferation was test by CCK‐8. (H) Western blot was used to evaluate ECM production of fibroblast. *p < 0.05

To explore the mechanism of Orai3 medicating Orai channel remodelling, we firstly assessed the expression levels of Orai3 protein. After the overexpression of Orai3, the no‐SOCE activity was slighted increased, and SOCE activity was weaken than NC group (Figure 5A,B). We also observed that after Orai3 overexpression, though the formation of Orai1 and Orai3 complex has increased (Figure 5C,D), but it is less than TGF‐β1 treated‐fibroblast (Figure 3C,D). These pointed the increased expression of Orai3 may not the main pro‐remodelling factor of Orai channel. So, we further assessed the formation of Orai1 and Orai3 complex under the condition not affected by increased Orai3 expression. The expression of flag linked to orai3 (flag‐Orai3) was expressed in fibroblast and was not affected by TGF‐β1 (Figure 5A), we were surprised to find TGF‐β1 induced increase of interaction of Orai1 and flag‐Orai3 (Figure 5E,F). Meantime, the fibroblast proliferation and ECM production was not increased in fibroblast with Orai3 overexpression but was enhanced in TGF‐β1‐treated fibroblast (Figure 5G,H). These data suggested that there are other mechanisms, not just relying on increase of Orai3 expression in Orai channel remodelling of TGF‐β1 administrated fibroblast.

### 3.6 CRACR2A and SEPTIN involved in fibrotic model in vivo and in vitro

**FIGURE 6:**
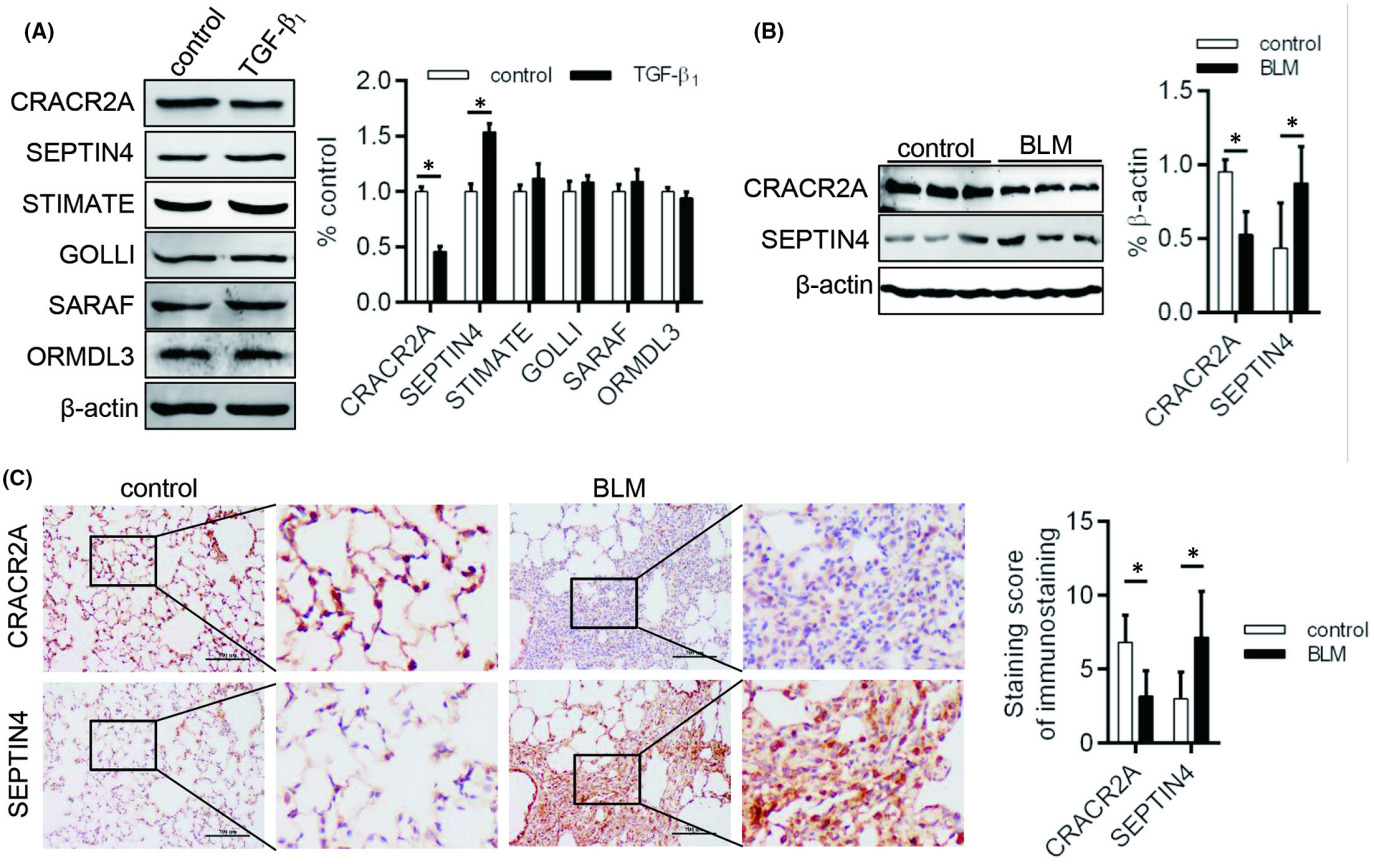
Expression of CRACR2A and SEPTIN in fibrotic model in vivo and in vitro. (A) Western blot was used to judge the change of CRACR2A, SEPTIN4, STIMATE, GOLLI, SARAF and ORMDL3 in TGF‐β1 treated‐fibroblast. The expression of CRACR2A and SEPTIN4 was detected by (B) Western blot and (C) immunohistochemistry. *p < 0.05

There are plenty of regulatory proteins, including CRACR2A, SEPTIN4, STIMATE, GOLLI, SARAF, ORMDL3 and so on, that contribute to alter SOCE activity in cancer, immune diseases and inflammation disorders.^16^, ^17^ To comfier these proteins whether involved in the formation of Orai1 and Orai3 complex to remodel Orai1 channel, we detected the expression levels of CRACR2A, SEPTIN4, STIMATE, GOLLI, SARAF and ORMDL3 in TGF‐β1 treated‐fibroblast and found that CRACR2A was significantly decreased and SEPTIN4 was obviously increased (Figure 6A). In addition, the decreased CRACR2A and increased SEPTIN4 were also found in lung from BLM‐induced rat (Figure 6B,C). These data showed that CRACR2A and SEPTIN may be associated with Orai1 channel remodelling of TGF‐β1 induced‐fibroblasts.

### 3.7 Orai1/Orai3 mediated TGF‐β1‐induced fibroblasts activation through SEPTIN4

**FIGURE 7:**
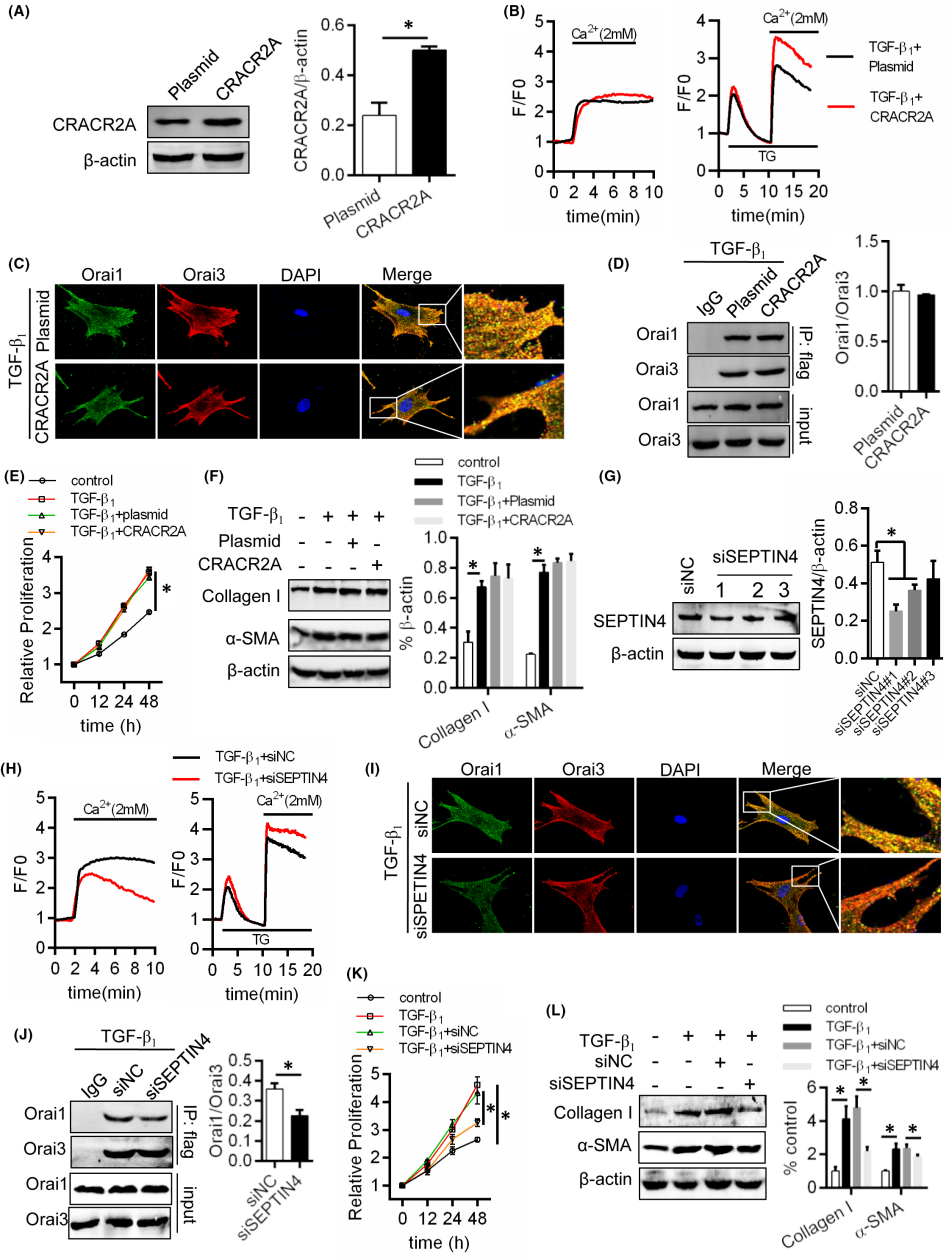
Effects of SEPTIN4 on the formation of Orai1 and Orai3 complex. (A) Plasmid carrying with CRACR2A was transfection into fibroblast to overexpress CRACR2A and then verified by Western blot. (B) Represented as Ca^2+^ peak amplitude in the F/F ratio over baseline values (F/F) after extracellular Ca^2+^ application for assessing no‐SOCE (left)/SOCE (right) activities. The formation of Orai1 and Orai3 complex was detected by (C) dual IF staining of Orai1 (green) and Orai3 (red) and (D) Co‐IP. Scale bars: 20 μm. (E) The potential of fibroblast proliferation was test by CCK‐8. (F) Western blot was used to evaluate ECM production of fibroblast. (G) Western blot was used to detect the expression of SEPTIN4 in fibroblast which was transfection siRNA and siSEPTIN4‐1 was used for following studies. (H) no‐SOCE (left)/SOCE (right) activities was compared between siSEPTIN4 and siNC group by flou‐4 staining. (I) Dual IF staining of Orai1 (green) and Orai3 (red) and (J) Co‐IP was performed for assessing the formation of Orai1 and Orai3 complex. Scale bars: 20 μm. (K) The potential of fibroblast proliferation was test by CCK‐8. (L) Western blot was used to evaluate ECM production of fibroblast. *p < 0.05

To determine whether CRACR2A and SEPTIN4 participate in Orai1 channel remodelling, we investigated these two proteins for further research, respectively. As CRACR2A was decreased in fibroblasts activation mentioned above (Figure 6), we overexpressed CRACR2A for further experiment (Figure 7A). The Ca^2+^ influx of no‐SOCE was not changed in fibroblasts with/without CRACR2A over‐expression (Figure 7B), and CRACR2A overexpression unable to do suppressed TGF‐β1‐induced formation of Orai1 and Orai3 complex, proliferation and ECM production (Figure 7C–F). We next focused on the association between the expression of SEPTIN4 and Orai1 channel remodelling. The expression of SEPTIN4 was knocked down by siRNA (Figure 7G) for its high expression in fibrotic model in vivo and in vitro (Figure 6). We surprisingly found that after knockdown, the expression of SEPTIN4, Ca^2+^ influx of no‐SOCE and SOCE was both significantly increased than fibroblast with siNC transfection and SOCE activity (Figure 7H). But TGF‐β1‐induced the formation of Orai1 and Orai3 complex obviously inhibited in fibroblast with SEPTIN4 knockdown (Figure 7I,J). Furthermore, SEPTIN4 knockdown also decreased TGF‐β1 induced‐cells proliferation and ECM production (Figure 7K,L). These data suggested that SEPTIN4 may be the main regulated factor for formation of Orai1 and Orai3 complex.

## 4 DISCUSSION

In this study, we found that the knockdown of Orai3 decreased TGF‐β1‐induced fibroblast proliferation, ECM production, activation of NFAT1 and Calpain/ERK signal pathway and glycolysis levels. TGF‐β1‐induced Orai3 interacting with Orai1 to remodel Orai channel and suppress SOCE activity, leading in a high and stable extracellular Ca^2+^ influx. And then, the deeper mechanism of increased SEPTIN4 induced formation of Orai1 and Orai3 complex.

As an important protein in classic SOCE, Orai1 has been widely concerned about its role in cell proliferation.^6^, ^18^ But some studied reported that elevated SOCE signals could drive cells into apoptosis.^19^, ^20^, ^21^ Let people re‐examine the role of the Orai protein family in cell proliferation. More studies focused Orai3 and found that Orai3 promoted cell proliferation and enhanced apoptosis resistance in tumour cells.^22^, ^23^ In our results, elevated levels of Orai3 were observed in lung fibrosis model in vivo and vitro. Additionally, the knockdown Orai3 could limit TGF‐β1‐induced proliferation and ECM production in fibroblast, and further mechanism might be related to the agitated nuclear translation of NFAT1, Calpain/ERK signal pathway and glycolysis, which were the mechanism of the fibroblasts activation.^24^, ^25^, ^26^ These suggest that Orai3 was implicated in the promotion of pulmonary fibrosis.

Orai3 is a calcium channel activated by Stim1 or Stim2,^9^ but in further studies, Orai3 interacted with Orai1 to form a heteromer was recognized in cancer cells for proliferation and decreased apoptosis.^7^, ^8^ We also found that the formation of Orai1 and Orai3 complex was increased fibrotic lung tissue and activated fibroblast. And high formation of Orai1 and Orai3 complex reduced the SOCE Ca^2+^ signalling, increased no‐SOCE Ca^2+^ signalling, leading to fibroblast proliferation and ECM production. However, it remains unclear how the different Ca^2+^ entry channel phenotypes determine the pro‐fibrosis switch. By measure the intracellular Ca^2+^ levels, the Orai channel remodelling enhanced basal and stable Ca^2+^ influx, and not the store‐dependent and undulated Ca^2+^ influx. Natalia Prevarskaya's research also supports that Orai channel remodelling‐mediated Ca^2+^ influx was an important mechanism for tumour cell proliferation and resistance to apoptosis, rather than SOCE.^8^ These data strongly suggest Orai channel remodelling‐regulated intracellular calcium stability may be an important internal environment for fibroblast proliferation.

Moreover, unlike other studies,^8^ we found that increased Orai channel remodelling was mainly does not rely on elevated levels of Orai3 protein. There are plenty of regulatory proteins, including CRACR2A, SEPTIN4, STIMATE, GOLLI, SARAF, ORMDL3 and so on, that contribute to alter Orai1 channel formation.^16^, ^17^ Our data shown that SEPTIN4 expression was increased and the crucial factor for Orai channel remodelling fibrotic modal. We also found that knockdown SEPTIN4 alleviated TGF‐β1‐induced proliferation and ECM production of fibroblast. In liver fibrosis studies, some literature stand by SEPTIN4 pro‐fibrosis,^27^, ^28^ but inhibiting fibrosis also was reported.^29^ It maybe need to further clarify the role and mechanism.

**FIGURE 8:**
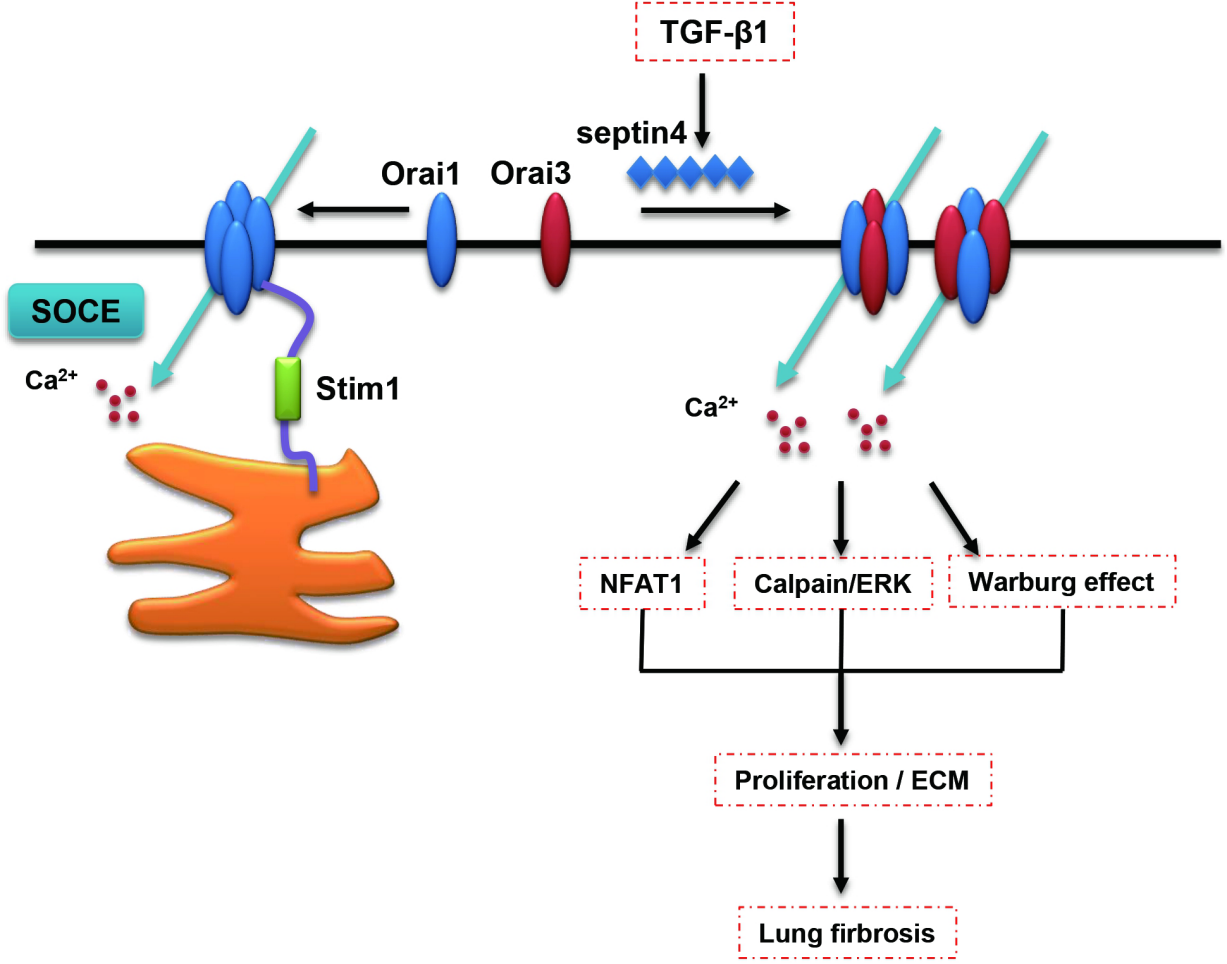
Schematic model for Orai3 mediate Orai channel remodelling to activate fibroblast in pulmonary fibrosis.

Altogether, our results found that Orai3 could interact with Orai1 to remodel Orai channel and suppress SOCE Ca^2+^ signal in fibrotic modal of lung and SEPTIN4 was the key regulatory proteins for Orai channel (Figure 8). That may establish a pro‐fibrotic role for the Orai3 and a mechanism via SEPTIN4 regulating Orai channel remodelling in pulmonary fibrosis. But how SEPTIN4 regulates the Orai channel remodelling is unclear, and we will further clarify in future research.

## AUTHOR CONTRIBUTIONS

**Changhui Yu:** Conceptualization (equal); data curation (equal); formal analysis (equal); funding acquisition (equal); investigation (equal); methodology (equal); resources (equal); writing – original draft (equal); writing – review and editing (equal). **Zicong Zhou:** Data curation (equal); formal analysis (equal). **Wufeng Huang:** Data curation (equal); investigation (equal); methodology (equal); writing – original draft (equal). **Xiumei Li:** Conceptualization (equal); data curation (equal); writing – original draft (equal). **Xiaojing Meng:** Writing – original draft (equal); writing – review and editing (equal). **Fei Zou:** Writing – original draft (equal); writing – review and editing (equal). **Shaoxi Cai:** Formal analysis (equal); funding acquisition (equal); project administration (equal); writing – review and editing (equal).

## CONFLICT OF INTEREST

The authors declare that they have no conflicts of interest.

## Supporting information


Figure S1

## Data Availability

The data that support the findings of this study are openly available in github at https://github.com/laoyu0001/changhui‐yu‐orai3.^30^
