# Supplementary material for: Orai3 mediates Orai channel remodelling to activate fibroblast in pulmonary fibrosis
Source: J Cell Mol Med. 2022 Sep 20;26(19):4974–85. doi: 10.1111/jcmm.17516 (PMC9549502; doi:10.1111/jcmm.17516)
Supplement: Supplementary file 1 — Figure S1 [file JCMM-26-4974-s001.zip › Supplement fig 1.docx]

**Supplement fig 1. The effects of Orai1 and Orai3 on no-SOCE and SOCE activities of fibroblasts.** The no-SOCE(lefet)/SOCE(right) activities was compared between Orai1 or/and Orai3 knockdown group and siNC group by flou-4 staining.
