# Supplementary figures and images for: Orai3 mediates Orai channel remodelling to activate fibroblast in pulmonary fibrosis
Source: J Cell Mol Med. 2022 Sep 20;26(19):4974–85. doi: 10.1111/jcmm.17516 (PMC9549502; doi:10.1111/jcmm.17516)

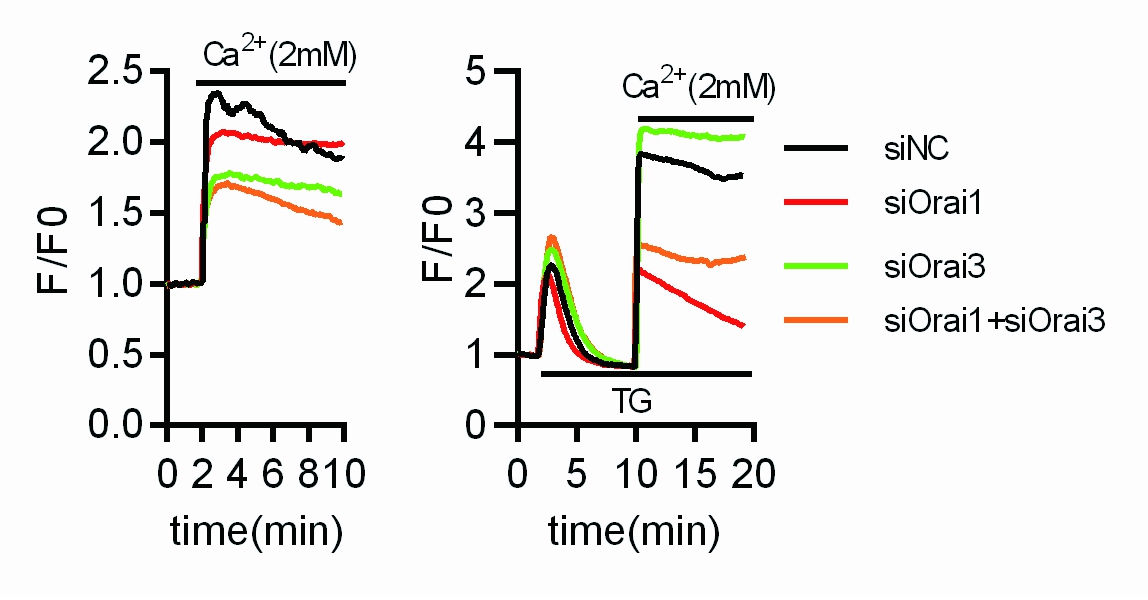

Supplement: Supplementary file 1 — Figure S1 [file JCMM-26-4974-s001.zip › JCMM_17516_fig S1.jpg]
